# A Retrospective Multicenter Study Identifies a Similar Overall Survival of Patients After Liver Transplantation With Incidental Cholangiocarcinoma Compared to Hepatocellular Carcinoma

**DOI:** 10.1111/ctr.70547

**Published:** 2026-05-19

**Authors:** Sophia Heinrich, Alejandro Campos‐Murguia, Angelina Mensah, Kateryna Shmanko, Dionysios Koliogiannis, Kathrin H. Vollmann, Simone C. Boedecker‐Lips, Bernd Heinrich, Theresa Kirchner, Bastian Engel, Christian M. Lange, Arndt Weinmann, Jens Mittler, Thomas C. Wirth, Anna Saborowski, Heiner Wedemeyer, Richard Taubert

**Affiliations:** ^1^ Gastroenterology Hepatology Infectious Diseases and Endocrinology Hannover Medical School Hannover Germany; ^2^ Department of Internal Medicine I University Medical Center of the Johannes Gutenberg‐University Mainz Mainz Germany; ^3^ Department of General Visceral and Transplantation Surgery Hospital of the University of Munich Ludwig‐Maximilians‐University München Germany; ^4^ Department of Internal Medicine I Hospital of the University of Munich Ludwig‐Maximilians‐University München Germany; ^5^ Department of General Visceral and Transplant Surgery University Medical Center of the Johannes Gutenberg‐University Mainz Mainz Germany

**Keywords:** chemotherapy, disease‐free survival, immunosuppression, intrahepatic cholangiocarcinoma, primary sclerosing cholangitis

## Abstract

**Background:**

Cholangiocarcinoma (CCA) is the second most common liver malignancy with a dismal prognosis. Liver transplantation of patients with CCA is currently no treatment option backed by guidelines for most of the patients, mainly because of presumably worse outcome. This study identified cases of incidental CCA (incCCA) in three German transplant centers, that is, CCAs unexpectedly found in liver explants, and analyzed the outcome in comparison to patients transplanted because of hepatocellular carcinoma (HCC).

**Methods:**

Histopathology reports of liver explants from patients transplanted between 2002 and 2021 in three transplant centers (Hanover, Mainz, and Munich) were screened. Cases of incidental biliary tract cancer were classified according to tumor node metastasis (TNM) staging and anatomic location. Overall (OS) and disease‐free survival (DFS) were analyzed and compared to patients transplanted due to HCC fulfilling the Milan criteria.

**Results:**

A total of 19 incCCA and 140 HCC patients were included in this analysis. IncCCA was a rare finding in liver explants and occurred most frequently in patients with primary sclerosing cholangitis (63%). Mean follow‐up of CCA patients was 61 (range, 4–254) months. Interestingly, OS and DFS of incCCA patients were not significantly different from patients transplanted with HCC (OS: *p* = 0.31; DFS *p* = 0.25). However, OS and DFS of incCCA patients differed significantly based on tumor location (OS HCC: N/A, intrahepatic CCA/perihilar CCA: 133 months, distal CCA/gallbladder: 6.5 months; *p* = 0.005; DFS HCC: 146 months, intrahepatic CCA/perihilar CCA: 133 months, distal CCA/gallbladder 5 months; *p* = 0.006).

**Conclusion:**

OS of patients diagnosed with incCCA was similar to HCC patients. These results add data to support transplantation of specific CCA patients as a reasonable treatment option.

**Abbreviations**
BTC: biliary tract cancer
CnI: calcineurin inhibitor
dCCA: distal cholangiocarcinoma
DFS: disease‐free survival
GBC: gallbladder cancer
HCC: hepatocellular carcinoma
iCCA: intrahepatic cholangiocarcinoma
incCCA: incidental CCA
LTR: liver transplant recipient
mTOR: mammalian target of rapamycin
OLT: orthotopic liver transplantation
OS: overall survival
pCCA: perihilar cholangiocarcinoma
TNM: tumor node metastasis

## 1 Introduction

Cholangiocarcinoma (CCA) is the second most common primary liver malignancy and carries a poor prognosis, as curative resection is feasible in only a minority of patients [1, 2, 3, 4, 5]. While liver transplantation (LT) achieves excellent outcomes in selected hepatocellular carcinoma (HCC) patients, CCA has historically shown inferior results. More recent data demonstrate improved outcomes in carefully selected CCA subgroups, highlighting the relevance of tumor biology and stage for transplant eligibility [6, 7, 8, 9, 10, 11, 12].

Selection for LT in malignant disease is constrained by donor organ scarcity and the oncological risks associated with long‐term immunosuppression. These factors necessitate strict prioritization based on expected post‐transplant outcomes. This is particularly evident in HCC, where exceptional MELD points and application of the Milan criteria have resulted in excellent outcomes, with reported 4‐year overall survival (OS) exceeding 75% and disease‐free survival (DFS) of approximately 83% [6, 13].

Incidental CCA (incCCA), defined as CCA unexpectedly detected in the explanted liver without radiologic suspicion before transplantation, represents a distinct and poorly characterized entity. IncCCA occurs predominantly in high‐risk populations such as patients with primary sclerosing cholangitis undergoing intensive surveillance, yet outcomes after LT remain incompletely described due to the rarity of this entity [14]. Existing data are largely limited to small retrospective series, and direct comparisons with established transplant indications are scarce.

In particular, outcomes of incCCA after LT remain poorly described, and no direct comparison to Milan‐criteria HCC when treated within the same healthcare systems and time periods has been performed in a multicenter German cohort. Addressing this gap is of high clinical relevance given ongoing debates regarding transplant eligibility for selected CCA subtypes.

This multicenter retrospective study evaluated OS and DFS in patients with incCCA identified on explant pathology after LT and compared outcomes with a contemporaneous cohort of patients transplanted for Milan‐criteria HCC. The analysis further explored whether outcomes differed by anatomic subtype and stage to identify incCAA phenotypes that may not have uniformly poor outcomes.

## 2 Material and Methods

### 2.1 Study Design

**FIGURE 1:**
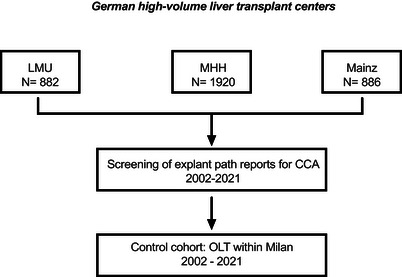
Study design. The flow chart outlines the screening process in this multicenter retrospective study.

We have retrospectively screened pathological explant reports for incCCA from liver transplant recipients (LTRs) at three German transplantation centers (Hannover, Mainz, Munich, see Figure 1). All participating centers were high‐volume liver transplant centers, performing approximately 40–70 LTs per year (annual DSO reports). Histopathological reports of liver explants from all patients undergoing LT between 2002 and 2021 were screened for the presence of biliary tract malignancies (Figure 1). incCCA was defined as histologically confirmed CCA detected in the explanted liver without radiologic or clinical suspicion before transplantation. No radiologic suspicion was defined as absence of any lesion interpreted pre‐transplant as suspicious for CCA requiring targeted diagnostic workup (e.g., mass‐forming lesion in contrast enhanced CT, MRI or ultrasound, progressive dominant stricture with malignant features, or imaging report explicitly raising concerns for CCA). All patients had undergone routine pretransplant imaging according to center‐specific protocols, including at least one cross‐sectional modality (contrast‐enhanced CT or MRI) and serial ultrasound examinations during listing as recommended by guidelines (for HCC within Milan criteria surveillance every 3 months, for CCA surveillance in PSC patients every 6–12 months). Surveillance intensity differed by indication and underlying liver disease, which may influence detectability of early lesions and constitutes a source of ascertainment bias. Imaging was performed within routine listing intervals; no standardized centralized imaging review was available due to the retrospective design. CA19‐9 levels have been assessed in period of 3 months prior LT. Patients transplanted for HCC within Milan criteria during the same period served as a comparison cohort. Because tumor node metastasis (TNM) editions evolved over the study period, T stage was harmonized at the level of T1 and T2 based on primary tumor extent as recorded in the original pathology reports. CCA was further categorized by anatomic location into intrahepatic (intrahepatic cholangiocarcinoma [iCCA]), perihilar (perihilar cholangiocarcinoma [pCCA]), distal (distal cholangiocarcinoma [dCCA]), and gallbladder cancer (GBC). Tumor surveillance was performed contrast‐enhanced CT or MRI after transplantation every 6 months for the first three years, and then yearly until year 5 after transplantation. Tumor recurrence was defined as radiologic evidence of recurrent disease on contrast‐enhanced CT or MRI, with histological confirmation when available. In cases without histological verification, recurrence was diagnosed based on characteristic imaging findings and clinical assessment. Recurrence‐free survival was calculated from the date of transplantation to the date of first radiologic or histologically confirmed recurrence or last follow‐up. Patients without recurrence were censored at last‐follow up. Death without documented recurrence was treated as an event for OS as well as for DFS. LTR were followed‐up until last contact or death (mean follow up for CCA were 61 months, for HCC were 74 months). Demographic and clinico‐pathological data were assessed for every patient (Table S1).

### 2.2 Pathological Assessment

Pathological assessment was performed locally at each center according to institutional standards. Data on tumor differentiation, resection margins, and lymph node status were extracted from pathology reports.

### 2.3 Ethics

This retrospective study was approved by the local ethical committee of the Hanover Medical School (No. 11363_BO_K_2024) and conducted in compliance with good clinical practice as well as in accordance with the Declaration of Helsinki. Patients included in this study gave informed consent. No organs or tissue were procured from prisoners.

### 2.4 Statistical Analysis

OS and DFS were estimated using Kaplan–Meier methods and compared using the log‐rank test. Given the limited sample size, analyses were primarily descriptive. Subgroup analyses were prespecified and interpreted descriptively without adjustment for multiplicity due to small sample size. Categorial variables were compared using Fisher's exact test due to small sample size.

Propensity score matching (PSM) was performed as an exploratory age at transplantation‐ and sex‐matched analysis. Matching was restricted to age at transplantation and sex using nearest‐neighbor matching in a 2:1 ratio because of limited overlap and missingness in additional covariates and to avoid overfitting/unstable estimates. Covariate balance before and after matching was assessed using standardized mean differences (SMDs) and visualized with Love plots (Figure S1C). An SMD <0.1 was considered indicative of adequate balance. PSM was not intended to enable causal inference, and no multivariable Cox regression was performed due to limited event numbers.

## 3 Results

### 3.1 Patient Baseline Characteristics

A total of 19 LTRs with incCCA could be identified over a period of 20 years at three high volume German transplantation centers (Hanover, Mainz, and Munich, Figure 1), performing 40–70 LT annually. This result corresponds to less than one case per center per year.

Patient baseline characteristics are shown in Table S1. A total of 140 LTR transplanted with HCC within Milan criteria served as a control. IncCCA patients were 68% male and 32% female. The dominant underlying liver disease was autoimmune liver disease (primary sclerosing cholangitis/autoimmune liver disease [63%]). The majority of incCCAs were intrahepatic (58%) followed by perihilar (21%). All tumors were staged as T1 or T2 according to TNM classification. The majority had a cellular differentiation of G1 or G2. 16% were classified as G3 (Table S1). Only one patient had resection status R1 after LT. Mean follow‐up time was 61 (range, 4–254) months. Median CA19‐9 level was 44 U/mL before LT, whereas 69% had normal levels, 31% of the patients had elevated levels of CA19‐9.

### 3.2 Overall Survival of Patients With Incidental CCA

**FIGURE 2:**
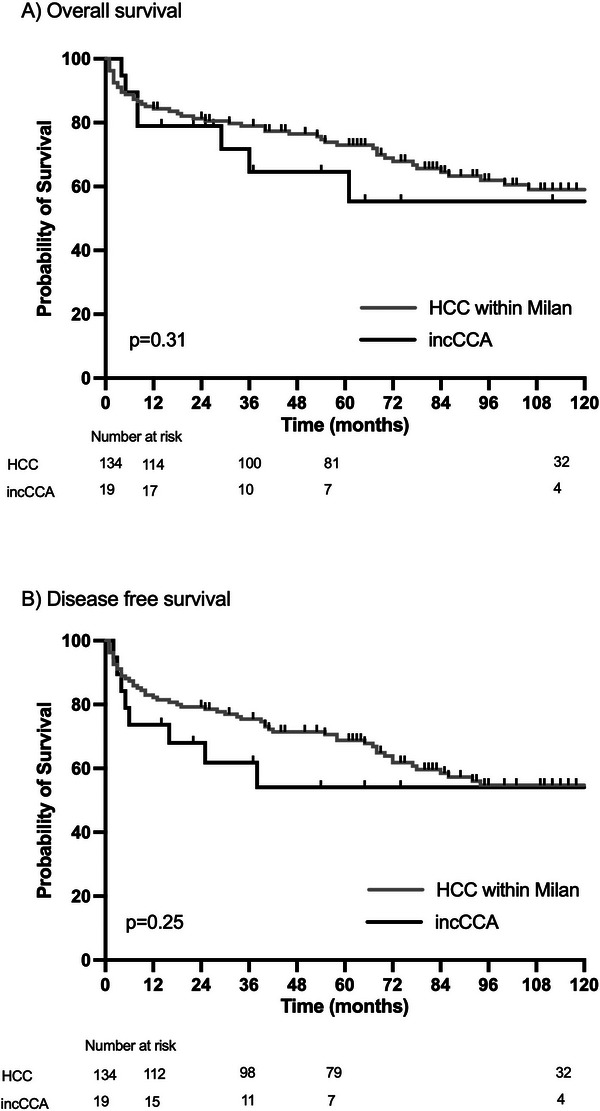
Survival of patients with incidental cholangiocellular carcinoma after liver transplantation. (A) Overall survival of patients with incCCA and patients with HCC transplanted Milan criteria. There is no significant survival difference (*p* = 0.31). (B) DFS of patients transplanted with HCC within Milan criteria and CCA patients. There is no significant difference in DFS of CCA patients than HCC patients (*p* = 0.25). CCA, cholangiocarcinoma; DFS, disease‐free survival; HCC, hepatocellular carcinoma; incCCA, incidental CCA.

We compared median OS of incCCA patients with HCC patients transplanted within Milan criteria. Median OS for incCCA patients was 133 months, and median was not reached for HCC patients. Importantly, there was no significant OS disadvantage for patients with incCCA compared to HCC patients ([*p* = 0.31], Figure 2). One, 3, and 5 years estimated survival rates for incCCA was 79%, 65%, and 65% respectively, which is comparable to HCC (84%, 79%, and 73%, respectively). In line with these results, DFS also did not differ significantly (Figure 2B, 5‐year DFS HCC 69%, CCA 54%, *p* = 0.26). Table S2 summarizes the distribution of tumor recurrence across the analyzed subgroups. Analysis of pretransplant CA19‐9 levels (available in 13 patients within 3 months before transplantation) showed that four patients had levels above the cutoff of 100 U/mL, while nine patients had values below this threshold. When comparing patients with and without tumor recurrence, all four patients with elevated CA19‐9 levels were in the recurrence group, whereas none of the patients without recurrence had elevated CA19‐9 levels (Table S3). This association was statistically significant (Fisher's exact test, *p* = 0.021)

To explore the robustness of the survival analyses, PSM was performed as a sensitivity analysis. Given the limited number of patients with incCCA and the incomplete availability or limited overlap of clinically relevant covariates between groups, matching was restricted to age at transplantation and sex as an explorative approach (Figure S2C).

In the matched cohort, OS did not differ significantly between groups (5‐year OS: HCC 69% vs. incCCA 55%; *p* = 0.49). Similarly, DFS remained comparable (5‐year DFS: HCC 69% vs. incCCA 54%; *p* = 0.31; Figure S1, patient cohort shown in Table S4). These findings were consistent with the results observed in the unmatched analysis.

### 3.3 Survival by Location and Stage of incCCA

**FIGURE 3:**
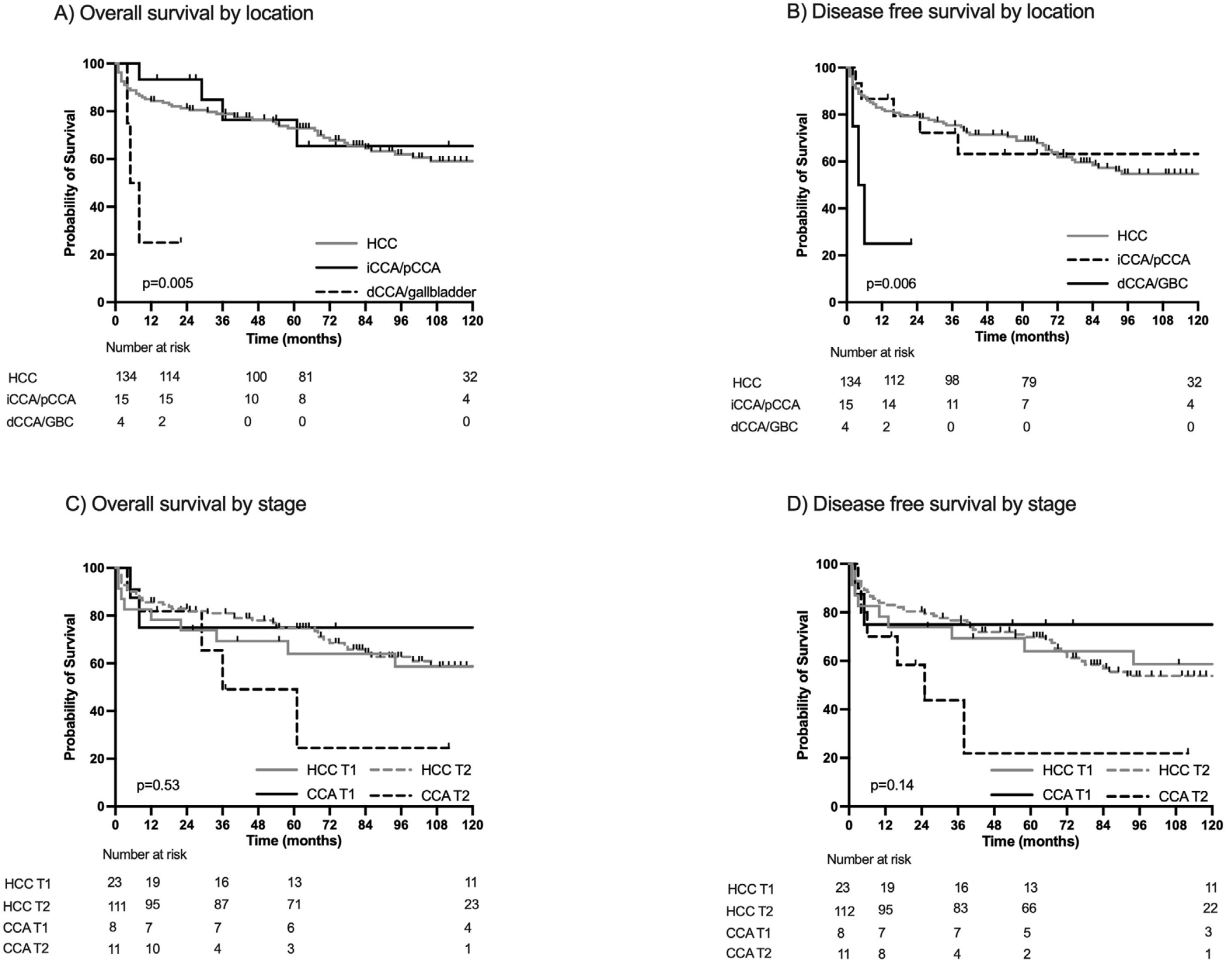
Survival of patients with incidental cholangiocellular carcinoma after liver transplantation according to tumor stage and localization. (A) OS of HCC and CCA patients stratified by tumor location. There is a significant survival benefit for iCCA/pCCA compared to dCCA/gallbladder cancer (*p* = 0.005), but no survival difference between HCC and iCCA/pCCA. (B) DFS by location. There is a significant difference in DFS by location (*p* = 0.006). There is an inferior DFS for dCCA/GBC cancer in this small cohort and no DFS benefit between HCC and iCCA/pCCA. (C) OS of HCC and CCA patients stratified by TNM stage. There is no significant survival benefit (*p* = 0.53). Survival estimates were numerically higher for T1 and T2; the comparison did not reach statistical significance. (D) DFS by stage. There is no significant difference in DFS by stage (*p* = 0.14). However, there is a trend for inferior DFS for stage T2 for incCCA in this small cohort. CCA, cholangiocarcinoma; dCCA, distal cholangiocarcinoma; DFS, disease‐free survival; GBC, gallbladder cancer; HCC, hepatocellular carcinoma; iCCA, intrahepatic cholangiocarcinoma; incCCA, incidental CCA; OS, overall survival; pCCA, perihilar cholangiocarcinoma; TNM, tumor node metastasis.

Since biliary tract cancer (BTC) is a heterogenous group of diseases, specifically differentiated by anatomical location, we have individually analyzed OS for anatomical subgroups of CCA grouping iCCA and pCCA as well as dCCA and GBC (Figure 3).

We observed differences in OS comparing dCCA/GBC and iCCA/pCCA, with dCCA/GBC showing a significantly worse survival within subgroups (Figure 3A, median OS HCC n/a, iCCA/pCCA 133 months, and dCCA/GBC 6.5 months, respectively, *p* = 0.005). Importantly, DFS comparing iCCA/pCCA subgroup with HCC patients did consistently not show any significant difference between HCC and iCCA/pCCA, while dCCA/GBC group showed again inferior DFS (Figure 3B, median DFS HCC 146 months, iCCA/pCCA 133 months, and dCCA/GBC 5 months, *p* = 0.006).

To determine, if there was a difference by stage, we performed a subgroup analysis of HCC and incCCA separated by extent of the tumor comparing TNM stage T1 and T2 (there were no higher stages than T2 in this cohort). A trend could be seen for OS with worse survival in T2 stage, not reaching significance (Figure 3C, median OS HCC T1 n/a, HCC T2 159 months, CCA T1 164 months, and CCA T2 36 months, respectively, *p* = 0.53). Tumor extent in patients with HCC does not significantly influence OS, with HCC stage T1 and T2 showing similar survival (HCC T1 vs. T2, *p* = 0.96). DFS analyses were performed and, similarly to OS, showed no significant difference but a trend for worse DFS for stage T2 CCAs (Figure 3D, median DFS HCC T1 n/a, HCC T2 141 months, CCA T1 133 months, and CCA T2 25 months, respectively, *p* = 0.14). One patient with dCCA presented with an R1 status following transplantation. To assess the potential impact of this case on our survival analyses, we performed a sensitivity analysis excluding this patient. The exclusion did not alter the results, and no significant difference in OS or DFS was observed (Figure S2A, B, median OS HCC n/a, CCA 133 months, *p* = 0.27; median DFS HCC 146 months, CCA 133 months, *p* = 0.42). Further, the impact of cellular differentiation as well as lymphonodal metastasis has been evaluated. There has not been a significant difference between groups (Figure S2C, D).

### 3.4 Influence and Strategies of Adjuvant Oncological Treatment

Next, we were interested in comparing different oncological treatment regimens of CCA patients after diagnosis of incCCA in the explant. The majority of patients (89.5%) did not receive any further adjuvant therapy (Table S5). OS of the subgroup without adjuvant therapy was 133 months (data not shown).

One patient received complementary chemoradiation (iCCA) and another received adjuvant gemcitabine/cisplatin (pCCA). IncCCA was recurrent in 32% of patients for the following locations: iCCA *N* = 3/12, pCCA *N* = 1/5, dCCA *N* = 1/3, and GBC *N* = 1/3. Both patients with adjuvant therapy did not show tumor recurrence. Recurrent disease occurred in different TNM stages (two patients initially classified as pT1, two as pT2 and two as pT2 and lymphonodal positive). In case of recurrence, the majority of patients (*N* = 3) received gemcitabine/cisplatin analogous to CCA patients outside the orthotopic liver transplantation (OLT) setting (Table S5). Equal numbers of patients received resection, radiotherapy, or chemoradiation (*N* = 1 each).

### 3.5 Influence of Immunosuppression

We further analyzed post‐transplant immunosuppressive regimens, as the choice of immunosuppression may transiently influence oncological outcomes after LT for HCC. We have focused on early calcineurin inhibitor (CnI) exposure and maintenance strategies at 6–12 months (Figure S3). In the early post‐transplant period (1–3 months), CnI trough levels could not be directly compared across all patients due to the lack of direct comparability between tacrolimus and cyclosporine levels and incomplete data availability. However, a descriptive overview of CnI exposure could be derived and is reported accordingly. In the HCC cohort, 78% of patients were within trough ranges of 5–8 ng/mL for tacrolimus and 875–100 ng/mL for ciclosporin, whereas in the CCA cohort, trough levels were unavailable in 45% of patients; among those with available data, 66% were in the respective tacrolimus or ciclosporin trough level (data not shown). Actual measured CnI trough levels as well as potential peak levels within the first 3 months were not available.

During maintenance immunosuppression (6–12 months), mammalian target of rapamycin (mTOR) was used in 21% of CCA patients compared with 5% of HCC patients. CnI‐based therapy remained the backbone of immunosuppression in both groups (CCA 70%, HCC 96%), with partial combination of CnI and mTOR regimes. Low‐dose steroid maintenance was observed in 16% of CCA patients and in 1% of HCC patients.

No consistent differences in immunosuppressive strategies were observed between patients with and without tumor recurrence (data not shown).

## 4 Discussion

BTC represents a rare but aggressive group of malignancies with limited curative treatment options. The diagnosis or secure exclusion of early‐stage CCA before LT remains challenging, particularly in high‐risk populations such as patients with PSC. Consequently, incCCA identified only on explant pathology is a rare disease but a clinically relevant finding after LT, as also reported by others [14].

In this multicenter retrospective study, we demonstrate that patients with incCCA have OS and DFS comparable to patients transplanted for HCC within Milan criteria. Importantly, this does not suggest that CCA in general performs similar to HCC, but rather that incCCA early‐stage iCCA/pCCA may represent a distinct biological subgroup with acceptable post‐transplant outcomes. By directly comparing incCCA with a contemporaneous HCC cohort within the same healthcare system and era, our study addresses a relevant knowledge gap.

Outcomes differed markedly by tumor location. Patients with intrahepatic or perihilar CCA demonstrated substantially better survival than those with distal CCA or GBC, supporting the concept that tumor biology and stage are critical determinants of outcome. These findings are consistent with registry data identifying tumor localization as a key prognostic factor and align with previous studies showing improved survival in selected CCA subgroups [14]. In particular, outcomes reported for perihilar CCA treated according to the Mayo protocol, with 5‐year survival rates of up to 80%, and for very early intrahepatic CCA exceeding 70%, underscore the importance of stringent selection and favorable tumor biology [7, 8, 9, 10, 11, 15, 16, 17, 18].

We observed no significant difference in OS or DFS between incCCA and HCC patients. This is clinically relevant, as HCC patients benefit from exceptional MELD points and achieve excellent outcomes, with reported 4‐year OS exceeding 75% and DFS of approximately 83% [6]. In contrast to Safdar et al. [14], who relied on historical reference data, our contemporaneous control cohort did not confirm inferior survival for incCCA. Tumor stage showed a non‐significant trend toward worse outcomes in T2 compared with T1 incCCA, consistent with prior reports [14]. Age and sex‐exploratory PSM supported these findings; given the small sample size and lack of overlap in clinically relevant variables, the matching approach should be interpreted as exploratory and does not fully account for potential confounding. The role of adjuvant oncological therapy after LT for incCCA remains unclear. Most patients in our cohort did not receive adjuvant treatment, reflecting the absence of standardized recommendations and earlier treatment era [17, 19, 20, 21]. Similarly, immunosuppressive regimens were heterogeneous in CCA patients, whereas HCC patients showed more uniform strategies, with increasing use of mTOR inhibitors over time, likely reflecting the influence of the SILVER trial [22]. However, CnI as the backbone of immunosuppression was the dominant immunosuppressive strategies over both tumor entities.

Several limitations warrant consideration. The rarity of incCCA resulted in a small sample size, limiting statistical power and precluding multivariable analyses. The retrospective comparison with HCC patients is inherently susceptible to immortal time bias due to differences in listing and surveillance. Pretransplant CA19‐9 levels showed a potential association with recurrence; however, given missingness and PSC‐related cholestasis, these findings should be interpreted as hypothesis‐generating [23, 24]. Given missingness and PSC‐related cholestasis, CA19‐9 findings should be interpreted as hypothesis‐generating.

In conclusion, incCCA after LT is rare but not uniformly associated with poor outcomes. Our data suggest that selected patients with intrahepatic or perihilar incCCA may achieve survival comparable to HCC within Milan criteria, supporting a more differentiated, biology‐driven perspective. Future efforts should focus on prospective registries, harmonized reporting, and refinement of selection criteria to better define the role of LT in CCA.

## Author Contributions

Sophia Heinrich, Heiner Wedemeyer, and Richard Taubert have contributed to conception and design of the paper. Sophia Heinrich, Angelina Mensah, Kateryna Shmanko, Dionysios Koliogiannis, Kathrin H. Vollmann, Simone C. Boedecker‐Lips, Bernd Heinrich, Theresa Kirchner, Bastian Engel, Christian M. Lange, Arndt Weinmann, Jens Mittler, Thomas C. Wirth, and Anna Saborowski have contributed collecting the data. Sophia Heinrich, Alejandro Campos‐Murguia, Thomas C. Wirth, Anna Saborowski, Heiner Wedemeyer, and Richard Taubert have analyzed and interpreted the data. Sophia Heinrich and Richard Taubert have written the manuscript. All authors have reviewed and agreed the final version of the manuscript.

## Funding

The work was supported by grants from the Integrated Research Center Transplantation funded by the German Federal Ministry lower Saxony of Education and Research and by the program of the voluntary scientific year. Bernd Heinrich was funded by the German Cancer Organization (Deutsche Krebshilfe). Bernd Heinrich, Dionysios Koliogiannis, and Christian M. Lange were supported by the Else Kröner‐Fresenius‐Stiftung. Bastian Engel was supported by the PRACTIS—Clinician Scientist program of Hannover Medical School, funded by the German Research Foundation (DFG, ME 3696/3).

## Conflicts of Interest

The authors declare that the research was conducted in the absence of any commercial or financial relationships that could be construed as a potential conflict of interest.

## Supporting information




**Table S1**: Patient cohort

**Table S2**: CA19‐9 levels before liver transplantation

**Table S3**: Distribution of tumor recurrence across the analyzed subgroups.

**Table S4**: Patient cohort after PSM

**Table S5**: Oncological treatment strategies. Overview of adjuvant oncological treatment administered after liver transplantation and treatement approaches in patients with tumor recurrence.

**Figure S1**: OS and DFS of HCC and CCA after explorative age at LT and sex‐PSM
A) OS of HCC and CCA patients after PSM. There is no significant survival benefit for HCC compared to CCA (p = 0.49). B) OS of HCC and CCA after PSM. There is no significant DFS benefit for HCC compared to CCA (p = 0.31). C) Covariate balance before and after PSM between patients with incCCA and HCC within Milan criteria. SMDs are shown for age at transplantation and sex before and after matching (age at transplantation: before matching ‐0.29, after matching 0.01 / gender: before matching 0.11, after matching 0.09). Values closer to zero indicate better balance, with |SMD| < 0.1 considered acceptable. PSM was performed as an exploratory sensitivity analysis and was restricted to age at transplantation and sex due to limited sample size and incomplete availability or overlap of additional covariates.

**Figure S2**: Survival of patients with incidental cholangiocellular carcinoma after liver transplantation according to tumor stage and grade
A) OS of HCC and CCA patients after exclusion of one patient with CCA and R1 status after OLT. There is no significant survival benefit for HCC compared to CCA (p = 0.27). B) DFS of HCC and CCA patients after exclusion of one patient with CCA and R1 status after OLT. There is no significant DFS benefit for HCC compared to CCA (p = 0.42). C) OS of HCC and CCA patients stratified by cellular differentiation (G). There is no significant survival benefit (p = 0.12). D) OS of HCC and CCA patients stratified by lymphonodal metastasis (N). There is no significant survival difference between groups (p = 0.14).

**Figure S3**: Distribution of immunosuppressive regimens during maintenance therapy (6–12 months) in patients with CCA and HCC. mTor inhibitors were used in 21% of CCA patients compared with 5% of HCC patients. CNI‐based therapy was applied in 70% of the CCA patients and 96% of HCC patients, with partial overlap between CNI and mTOR‐based regimens. Low‐dose steroid maintenance was observed in 16% of CCA patients and 1% of HCC patients.

## Data Availability

The authors have nothing to report
