# Supplementary material for: A Retrospective Multicenter Study Identifies a Similar Overall Survival of Patients After Liver Transplantation With Incidental Cholangiocarcinoma Compared to Hepatocellular Carcinoma
Source: Clin Transplant. 2026 May 19;40:e70547. doi: 10.1111/ctr.70547 (PMC13185673; doi:10.1111/ctr.70547)

Supp. Figure 1: OS and DFS of HCC and CCA after PSM, Love plot

A) Overall survival

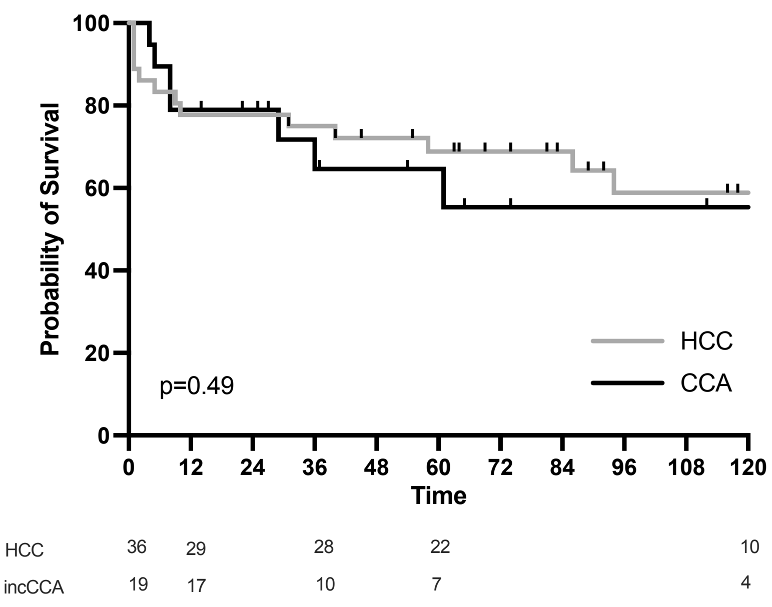

B) Disease free survival

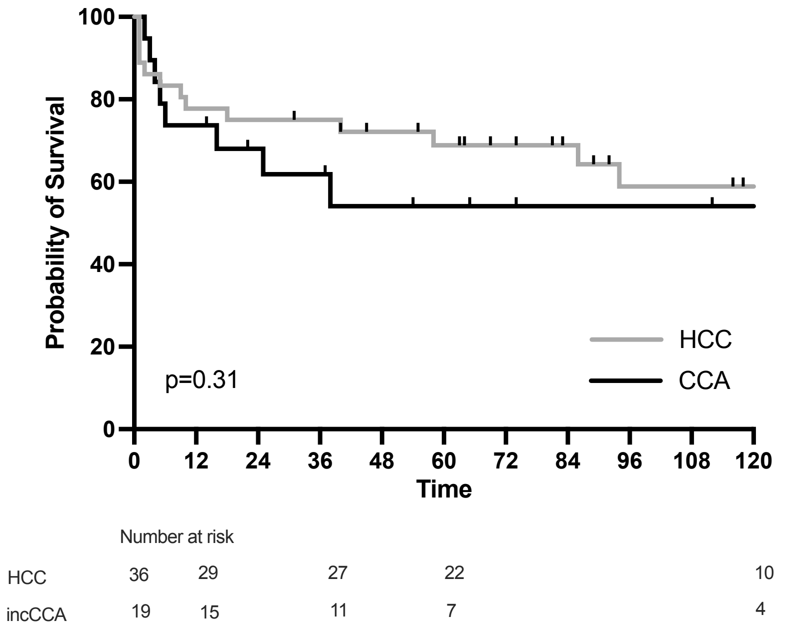

C) Love plot

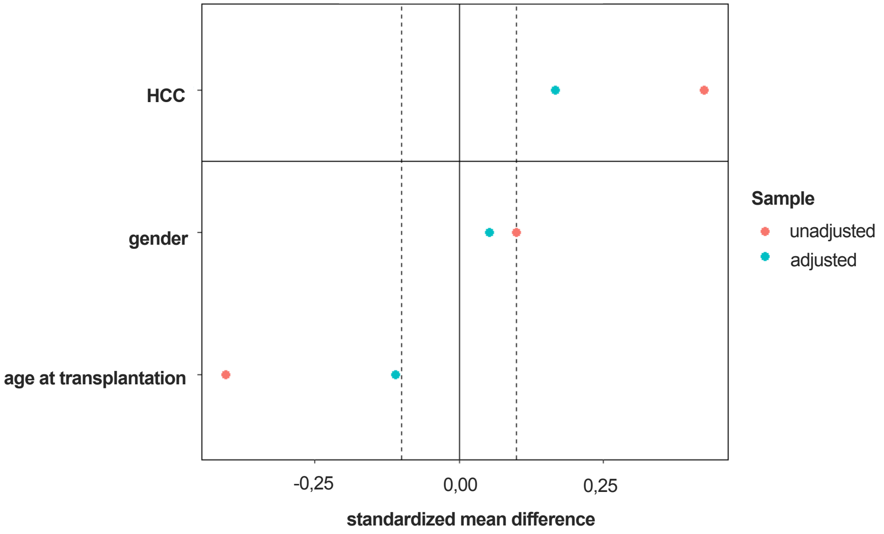

Supplement: Supplementary file 6 — Figure S1: OS and DFS of HCC and CCA after explorative age at LT and sex‐PSM A) OS of HCC and CCA patients after PSM. There is no significant survival benefit for HCC compared to CCA (p = 0.49). B) OS of HCC and CCA after PSM. There is no significant DFS benefit for HCC compared to CCA (p = 0.31). C) Covariate balance before and after PSM between patients with incCCA and HCC within Milan criteria. SMDs are shown for age at transplantation and sex before and after matching (age at transplantation: before matching ‐0.29, after matching 0.01 / gender: before matching 0.11, after matching 0.09). Values closer to zero indicate better balance, with |SMD| < 0.1 considered acceptable. PSM was performed as an exploratory sensitivity analysis and was restricted to age at transplantation and sex due to limited sample size and incomplete availability or overlap of additional covariates. [file CTR-40-e70547-s008.pdf]
