# Supplementary figures and images for: A Retrospective Multicenter Study Identifies a Similar Overall Survival of Patients After Liver Transplantation With Incidental Cholangiocarcinoma Compared to Hepatocellular Carcinoma
Source: Clin Transplant. 2026 May 19;40:e70547. doi: 10.1111/ctr.70547 (PMC13185673; doi:10.1111/ctr.70547)

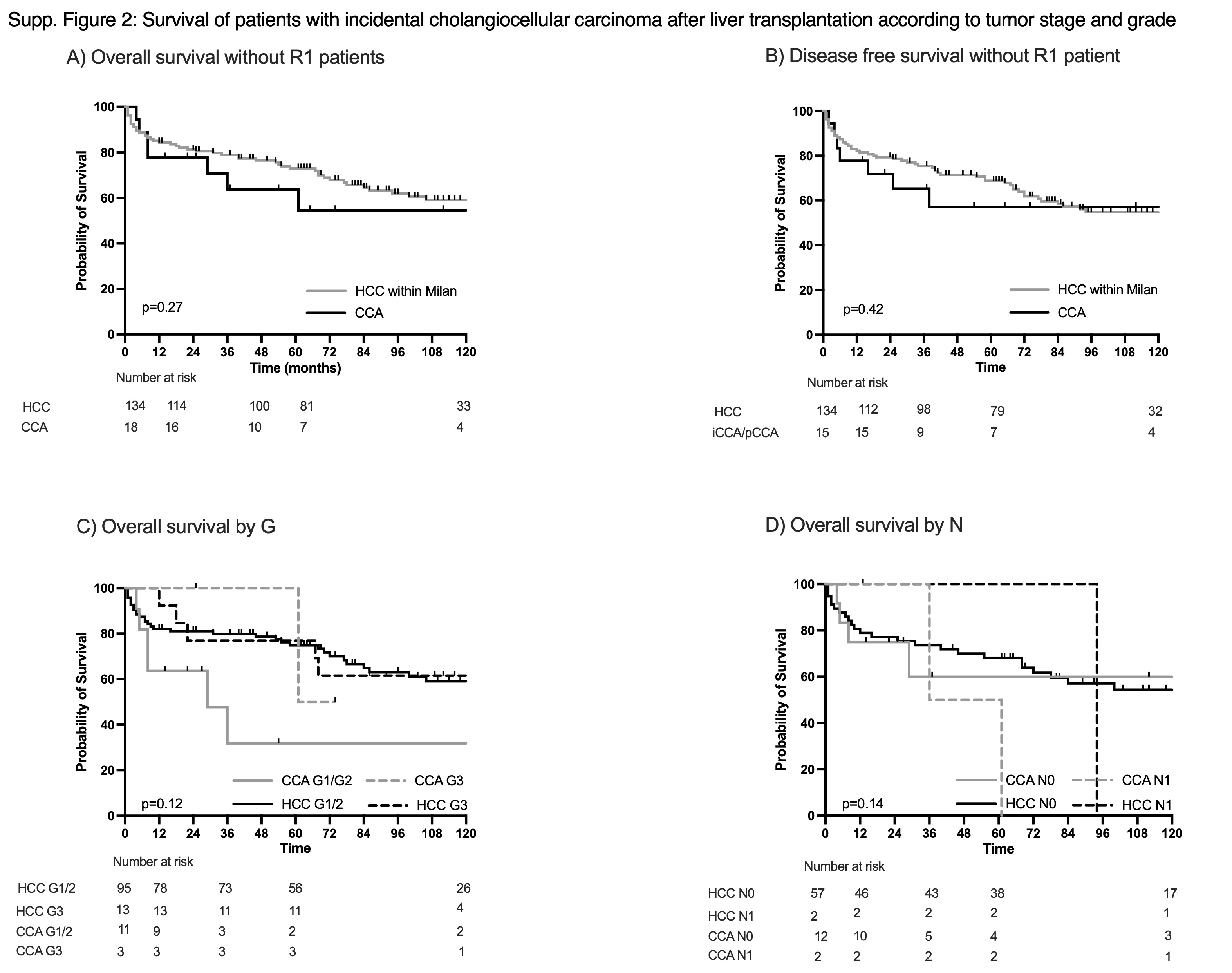

Supplement: Supplementary file 7 — Figure S2: Survival of patients with incidental cholangiocellular carcinoma after liver transplantation according to tumor stage and grade A) OS of HCC and CCA patients after exclusion of one patient with CCA and R1 status after OLT. There is no significant survival benefit for HCC compared to CCA (p = 0.27). B) DFS of HCC and CCA patients after exclusion of one patient with CCA and R1 status after OLT. There is no significant DFS benefit for HCC compared to CCA (p = 0.42). C) OS of HCC and CCA patients stratified by cellular differentiation (G). There is no significant survival benefit (p = 0.12). D) OS of HCC and CCA patients stratified by lymphonodal metastasis (N). There is no significant survival difference between groups (p = 0.14). [file CTR-40-e70547-s006.jpg]

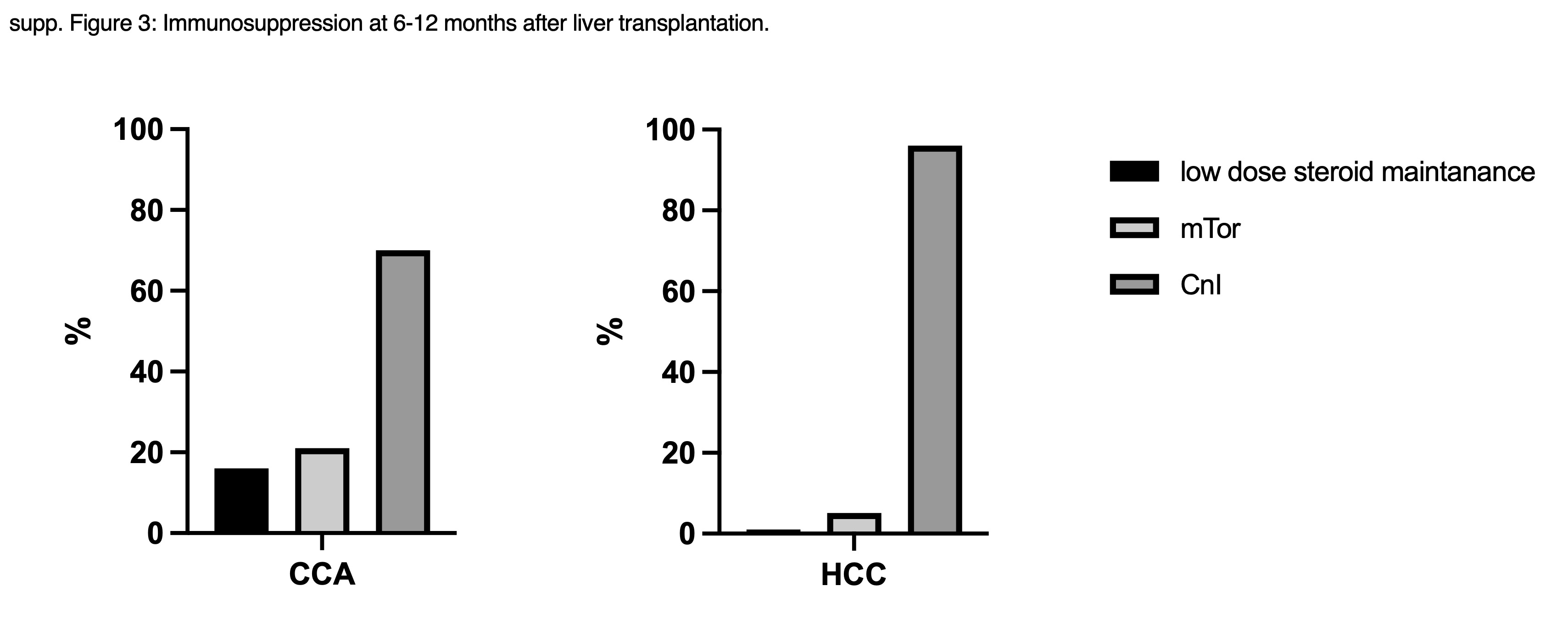

Supplement: Supplementary file 8 — Figure S3: Distribution of immunosuppressive regimens during maintenance therapy (6–12 months) in patients with CCA and HCC. mTor inhibitors were used in 21% of CCA patients compared with 5% of HCC patients. CNI‐based therapy was applied in 70% of the CCA patients and 96% of HCC patients, with partial overlap between CNI and mTOR‐based regimens. Low‐dose steroid maintenance was observed in 16% of CCA patients and 1% of HCC patients. [file CTR-40-e70547-s001.jpeg]
